# Uterine tumor resembling ovarian sex cord tumor: A rare case report

**DOI:** 10.1097/MD.0000000000030414

**Published:** 2022-09-02

**Authors:** Xue-Ying Wang, Mao-Chun Zhang, Jiao Chen, Jiang-Hua Huang

**Affiliations:** a Department of Ultrasound, Affiliated Hospital of North Sichuan Medical College, Nanchong, Sichuan ProvinceChina; b Department of Obstetrics and Gynecology, Affiliated Hospital of North Sichuan Medical College, Nanchong, Sichuan ProvinceChina; c Department of Ultrasound, the Second Clinical Medical College of North Sichuan Medical College, Nanchong, Sichuan ProvinceChina.

**Keywords:** case report, immunohistochemical, treatment, uterine tumor resembling ovarian sex cord tumor

## Abstract

A uterine tumor resembling an ovarian sex cord tumor (UTROSCT) is a clinically rare disease with an unclear origin and biological behavior.

**Patient concerns:**

We present a case of UTROSCT in a 42-year-old woman who presented with abnormally increased menstrual volume for 2 years.

**Diagnoses:**

Initially, only ultrasound examination was performed to diagnose uterine fibroids, and then the tumor was surgically removed and sent for pathological examination. The patient was ultimately diagnosed with UTROSCT mainly based on pathological immunohistochemical examination and was further diagnosed with low malignant potential for recurrence based on genetic testing.

**Interventions and Outcomes:**

The patient underwent hysterectomy and bilateral adnexectomy, and no adjuvant radiotherapy or chemotherapy was performed after the surgery. Follow-up to date has indicated that she is in good condition.

**Lessons:**

UTROSCT is a rare disease that requires pathological immunohistochemical examination to confirm the diagnosis and genetic testing when necessary so that a clear diagnosis can inform better decision-making regarding treatment measures.

## 1. Introduction

Uterine tumor resembling an ovarian sex cord tumor (UTROSCT) is a relatively rare disease. It tends to occur in perimenopausal and postmenopausal women and is rare in young people under the age of 25. The main manifestations of UTROSCT are abnormal vaginal bleeding, increased menstrual volume, lower abdominal distension, pelvic pain, or no manifestations. Here, we reported a case of UTROSCT; the patient was admitted to the second Clinical Medical College of North Sichuan Medical College. Initially, the patient only underwent tumor resection, and the specimen was sent to West China Hospital of Sichuan University for pathological consultation and genetic testing; thus, the patient was further diagnosed as having a low possibility of malignant recurrence.

## 2. Case presentation

**Figure 1.**
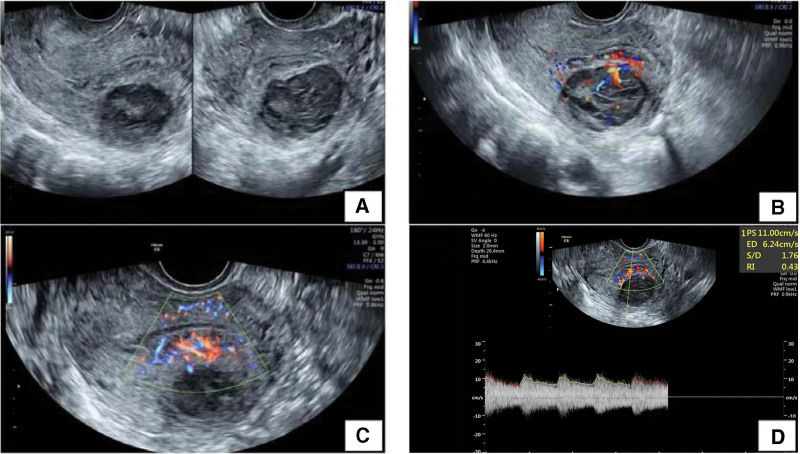
(A, B) A hypoechoic mass (3.9 cm × 3.2 cm × 3.9 cm) was observed in the posterior wall of the uterus. (C, D) A hypoechoic mass (4.6 cm × 1.1 cm × 3.7 cm) was observed on the uterine endometrium. (A–D) The 2 masses were not well demarcated. (D) Blood flow spectrum, (PS: 11.00 cm/s; S/D: 1.76: RI: 0.43).

**Figure 2.**
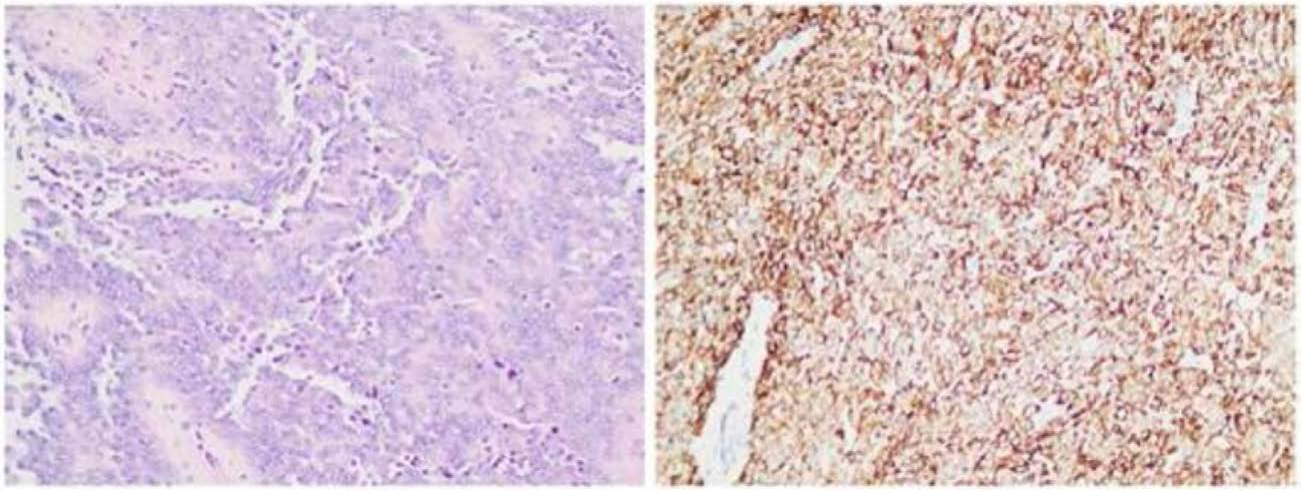
(Left) The mass specimen showed proliferative intrauterine endometrium and short spindle cells diffuse (H&E, ×100). (Right) immunocytochemistry. H&E = hematoxylin and eosin.

A 42-year-old woman came to see us complaining of abnormally increased menstrual volume for 2 years. She had a nodule removed from her breast 2 years prior, and her mother had breast cancer, but she denied a family history. Three years ago, during a checkup, a lump was found on the uterus and was diagnosed as a uterine fibroid based on ultrasound but was ignored. One year later, she started to have increased menstrual volume. At that time, we advised her to have surgery to remove the fibroid, but she refused surgery for personal reasons. Two months ago, she came here for ultrasound review (Fig. 1), and low-echoic lumps were found in both the posterior wall of the uterus and the endometrium (the 2 masses were not well demarcated); the ultrasound diagnosis was uterine fibroid and uterine submucosal fibroid. We again recommended that she undergo surgery, and she agreed. She also provided written informed consent to publish this case report. We performed laparoscopy and hysteroscopy on the membranes for removal of her uterus and intrauterine lesions and resected the mass during the operation process to generate frozen sections for diagnosis. Microscopically, the results suggested a proliferative intrauterine membrane with a diffuse distribution of short spindle cells, suggesting neoplastic lesions. After the operation, we sent all the specimens for pathological examination (Fig. 2) and obtained the same result, namely, that the proliferative endometrial membrane and tumor showed invasive growth in the muscular layer of the uterine wall. The immunohistochemical staining results were as follows: Desmin (+), SMA (focal+), H-caldesmon (–), CD10 (–), CD117 (–), CyclinD1 (–), CD99(+), WT-1 (+), D2-40 (+), CR (–), a-inhibin (–), MyoD1 (–), Melan A (–), and Ki-67 (Hot spot+, approximately 10%). These findings, in combination with hematoxylin–eosin staining morphology and immunophenotype, suggested UTROSCT. Then, the specimen was sent to West China Hospital of Sichuan University for pathological consultation and genetic testing, which indicated that no translocation of the *JAZF1* gene was detected, and the tumor had an unclear boundary with the muscle wall and involved the endometrium, with low malignant potential for recurrence. Therefore, we removed her entire uterus and both fallopian tubes laparoscopically again, and she was doing fine without adjuvant chemoradiotherapy after the operation.

## 3. Discussion

UTROSCT is a relatively rare disease. In 1976, Clement and Scully first reported this tumor, describing it as a tumor with a large sex cord component (>50%) but little or no stromal component, and its morphology was similar to that of ovarian sex cord tumors.^[1]^ In 2020, the World Health Organization changed the classification of female genital tumors, defining UTROSCT as a uterine tumor similar in shape to ovarian sex cord tumors and further clarifying that there is no discernible endometrial stromal component in this tumor tissue.^[2]^ At present, the etiology of UTROSCT is not clear and may be related to mesenchymal cell pluripotent differentiation,^[3]^ ovarian sex cord cells, or ectopic interstitial components of the endometrium.^[2]^ It has also been suggested that the occurrence of UTROSCT may be related to the use of tamoxifen.^[4]^ In the course of diagnosis and treatment of our patient with UTROSCT, the importance of understanding the key points of clinical diagnosis and treatment of this disease has been reaffirmed for us.

First, doctors had the idea of surgical treatment when they discovered that the patient had a mass in her uterus and an abnormal increase in menstrual volume. Generally, observation and follow-up are recommended for patients when their daily life is not affected. However, in this case, the abnormal increase in menstrual volume and the constant presence of the mass had begun to cause dizziness and other discomforts in the patient. We considered the possible cause to be slightly more blood loss, so we still suggested that the patient undergo surgery to remove the mass.

Second, the boundaries between the low echo crumb in the back wall of the uterus and the low echo crumb in the uterine membrane can be indistinguishable on ultrasound, suggesting that the 2 are fused or come from the same group. Ultrasound, computed tomography, and magnetic resonance imaging can all be used to examine UTROSCT.^[5,6]^ However, due to the rarity of the disease in clinical practice and insufficient data, there is no unified standard diagnosis on imaging, and imaging cannot confirm the diagnosis. At present, the diagnosis of UTROSCT depends mainly on pathological tissue examination, immunohistochemistry, and other related auxiliary examinations. Therefore, we first removed the patient’s tumor, subjected the tumor tissue to pathological examination, conducted genetic testing for further diagnosis, and finally diagnosed UTROSCT with low malignant potential.

Third, the effect of adjuvant therapy on UTROSCT is still unclear. One study^[7]^ found no difference in the outcome of a follow-up study of a patient who received pelvic and vaginal brachytherapy compared with a patient who did not receive radiotherapy. Then, to prevent the poor prognosis associated with tumor recurrence and metastasis, we removed the entire uterus and both fallopian tubes of the patient without postoperative adjuvant therapy, and she is now in good condition.

However, it is worth noting that UTROSCT is almost entirely made up of sex cord components, and most uterine lesions can also have sex cord-like components, among which endometrial stromal tumors are the most common, and the prognosis of these endometrial tumors is significantly poorer than that of UTROSCT.^[8]^ Therefore, the differential diagnosis of these tumors is particularly important. UTROSCT does not contain endometrial stromal components. CD10, a specific marker of endometrial stromal components,^[9]^ is often negative in UTROSCT, which is consistent with this case. At the same time, JAZF1/SUZ12 fusion (characteristic of sex cord-like differentiated endometrial stromal sarcomas) was not found in UTROSCT,^[10]^ as confirmed by genetic testing in this case.

Finally, although UTROSCT is mostly benign, it still has malignant potential, and the feasible treatment is whole uterus removal with or without bilateral adnexectomy and postoperative follow-up. However, if the patient has the need for fertility, a lump alone can be resected first, with close follow-up after the operation, and hysterectomy is recommended after delivery. Adjuvant radiotherapy and chemotherapy are generally not performed after the operation, but for patients with local infiltration or distant metastasis, postoperative adjuvant radiotherapy and chemotherapy are recommended.

UTROSCT is rare. Here, we have reported our complete diagnosis and treatment insights regarding UTROSCT based on this case. In line with the principle of being responsible for patients, we need to understand the key points of clinical diagnosis, differential diagnosis, and treatment of this disease and affirm that a clear diagnosis can better inform decision-making regarding treatment measures.

## Author contributions

Xueying Wang, Maochun Zhang, Jiao Chen, Jianghua Huang identified the medical and scientific significance of this case. Xueying Wang, Jiao Chen and Jianghua Huang collected and analyzed these clinical data. Xueying Wang wrote the manuscript. Xueying Wang, Maochun Zhang and Jiao Chen revised the manuscript. And all authors read the final manuscript.
